# The use of cash assistance in the Covid‐19 humanitarian response: accelerating trends and missed opportunities

**DOI:** 10.1111/disa.12524

**Published:** 2021-12-07

**Authors:** Julie Lawson‐McDowall, Ruth McCormack, Sophie Tholstrup

**Affiliations:** ^1^ Technical Advisor CALP Network United Kingdom; ^2^ Technical Advisor CALP Network Canada; ^3^ Former Policy Coordinator CALP Network Switzerland

**Keywords:** cash and voucher assistance (CVA), Covid‐19, humanitarian assistance, social protection

## Abstract

The operational and socioeconomic consequences of Covid‐19 have made cash assistance the global go‐to relief modality, whether through humanitarian or social protection channels. Cash has proven to be an adaptable means of saving lives and supporting livelihoods and mitigating the pandemic's impacts on local economies while giving recipients the flexibility to decide what they require. Many humanitarian organisations have increased the scale of cash programmes, while government‐administered social assistance mechanisms have been utilised on a huge scale. The crisis has bolstered attention on why linkages between social protection and humanitarian cash are important, including how to work together more effectively to enable better coverage of those in need. This paper has been developed with inputs from across the CALP Network. It explores how cash and voucher assistance—with a focus on humanitarian response—has been scaled up or adjusted in response to Covid‐19, and how it is changing ways of working.

## Introduction

The Covid‐19 pandemic transformed the way in which humanitarian assistance is delivered almost overnight. In a sector often resistant to change, the pace of adaptation—both to a constrained operating environment and to rising economic needs— has been extraordinary. Globally, cash and voucher assistance (CVA) has proven to be an appropriate and adaptable means of addressing essential needs and supporting livelihoods affected by the pandemic through humanitarian channels, government social assistance, or some combination of the two. This paper looks at how CVA has been adapted in (mostly) humanitarian contexts during the pandemic and what these changes may mean for programming in the future.

This crisis has precipitated an increase in extreme poverty for the first time in 20 years, with the World Bank estimating that an additional 124 million people may be living on less than USD 1.90 per day by the end of 2021 (Mahler et al., 2021). One study of the impacts in low‐ and middle‐income countries found that a median of 70 per cent of respondents had experienced a drop in income, with many unable to meet basic nutritional needs (IPA, 2020). In contexts affected by ongoing humanitarian crises, the pandemic has often compounded existing needs and funding has remained well below what is required.^1^

The scaling up of humanitarian cash assistance in recent years has been significant, with CVA increasing from USD 2.8 billion in 2016 to USD 6.3 billion in 2020, constituting one‐fifth of international humanitarian assistance (Development Initiatives, 2021). From the outset of the pandemic, feedback from humanitarian agencies and Cash Working Groups (CWGs) indicated an expansion of CVA in response to the economic impacts of Covid‐19 (CALP Network and CashCap, 2020). Analysis shows that the volume of CVA delivered to recipients grew by 16 per cent between 2019 and 2020, at least partially because of the pandemic response. Following a 25 per cent increase in CVA from 2019 to 2020, non‐governmental organisations (NGOs) described it as a central component of their responses to Covid‐19 (Development Initiatives, 2021). For example, World Vision International recorded a rise in the volume of CVA of approximately 28 per cent between 2019 and 2020 (World Vision, 2020a, 2020b), while the International Rescue Committee in Yemen augmented its cash assistance caseload by 30 per cent between March and September 2020 (IRC, 2021).

The delivery of social protection as cash transfers is well established, but the pandemic saw a huge upturn in scale: ‘almost 17% of the world's population has been covered with at least one Covid‐related cash transfer payment between 2020 and 2021’ (Gentilini, 2021b; Smith, 2021). However, the value and duration of support has been highly variable, with minimal to no government provision in some fragile and/or humanitarian settings. In many such contexts, humanitarians have filled the gap or have worked with governmental and developmental actors to strengthen responses through a creative blend of social protection and humanitarian approaches (Longhurst et al., 2020; Beazley, Bischler, and Doyle, 2021; Smith, 2021; see also Interview, Barca, 2021).^2^

This paper draws on a mixture of primary and secondary data, comprising 24 key informant interviews with CALP Network members (see the Appendix for a complete list), and a non‐comprehensive literature review. It is important to acknowledge the formative methodological role of this crowdsourced interview data, primarily from implementers and funders of humanitarian CVA, in shaping this paper. The use of CVA in the pandemic is a very broad topic, and this paper focuses on the most relevant findings of our research, organised by five themes:

- how cash has proven to be a suitable and rapid response tool in the pandemic;
- remote programming and the intensified digitalisation of CVA;
- the expansion of social protection and linkages with the humanitarian response;
- exploration of whether localisation has been a missed opportunity; and
- an examination of perspectives on the potential long‐term changes to CVA owing to Covid‐19.

## Cash has been an adaptable and rapid response tool in the pandemic

The onset of the pandemic presented humanitarian organisations with the multi‐faceted challenge of scaling up programming in light of rising needs and adjusting to new ways of working, while maintaining the quality of programmes and ensuring that communities and staff members were kept as safe as possible (Interview, Nawar, 2021; Interview, Toscano, 2021). CVA emerged as an effective mode of assistance, lending itself to remote delivery and supporting recipients in addressing varied needs. As organisations raced to adapt, they were guided by the swift production of multiple new tools and guidance (see, for example, Golay, 2020). This section summarises some of the key factors informing the use of CVA in the pandemic response.

### Cash has been a preferred form of assistance

Data gathered during the pandemic continues to show that people in crisis generally prefer cash, and that it is an effective way of meeting needs. For instance, the United Nations Refugee Agency (UNHCR)'s analysis of post‐distribution monitoring data found that 80 per cent of respondents preferred cash assistance and that 88 per cent could locate what they needed in the market (UNHCR, 2020). Similarly, data from Colombia in early 2021 revealed that between 72 and 80 per cent of households preferred cash assistance, depending on which needs (such as food or shelter) were most important to them (R4V, 2021, cited in IFRC, 2021). This is not a universal finding, however, and is influenced by contextual factors. In Nigeria, Ground Truth Solutions (2020b) found significant variations in modality preference during 2020, with in‐kind and/or vouchers preferred in some places, largely because of reduced spending power due to inflation, currency devaluations, and trade restrictions.

### Markets largely functioning and accessible, with limitations

Effective CVA requires functional and accessible markets. The restrictions imposed by governments to reduce the spread of Covid‐19 (Hale et al., 2021) have variously affected market systems, supply chains, and trade. One study discovered that 30 per cent of respondents had experienced reduced market access (IPA, 2020). But even when access is highly constrained, markets have generally been able to adapt and continue. In Burkina Faso, it was reported that ‘market systems resisted and continued to be a main source for basic goods’ (Interview, Amougou, 2021). In Myanmar, market feasibility studies undertaken by Plan International in camps for displaced people found that most were still functional and able to meet local needs and had relatively stable access to food (Alam, 2020).

### Market monitoring and programme adaptations

The impacts on supply chains, access, and prices (CALP Network, 2020) meant that market monitoring became increasingly vital, with agencies stepping up related activities. Examples include the increased frequency of market surveys by the Cash Consortium of Iraq, complementing data on community perspectives on market and socioeconomic conditions (Leape and Zasadowska, 2021). In Syria, adjustments were made to ensure commodities relevant to the Covid‐19 response were included in market monitoring, led by REACH. This helped to identify shortages and price hikes, enabling agencies to determine when mixed and alternative assistance modalities would be required (Interview, Wynne, 2021).

### Donor support and flexibility

Critically, donors have supported the scaling up of cash assistance during the pandemic. The members of the Donor Cash Forum produced a set of recommendations for engaging with partners, including better use of digital solutions and stronger links with social protection mechanisms (Donor Cash Forum, 2020). ECHO (European Civil Protection and Humanitarian Aid Operations) was among the donors that ‘sent a clear message to partners that scaling up cash further and adaptations to existing cash programmes were actively encouraged’ (Interview, Pelly, 2021). The Government of the United States’ Bureau of Population, Refugees, and Migration (PRM) retains a ‘modality neutral’ approach to programming: while not specifically advocating with partners to increase CVA, it has, anecdotally, observed a rising number of partners using it (Interview, PRM, 2021). At the field level, there is evidence of donors supporting the need for budget flexibility to accommodate additional costs due to Covid‐19 safety protocols. In Zimbabwe, for instance, agencies were successful in jointly advocating donors in this regard via the Humanitarian Country Team (Interview, Prandini, 2021). Studies of adaptation of CVA to crises in Jordan and Lebanon in 2020 noted that donor flexibility in supporting local decision‐making by humanitarian actors was critical (Dürr, 2021).

### Expanding coverage

Organisations rapidly expanded CVA coverage in response to Covid‐19. Examples include the Democratic Republic of the Congo (DRC), which recorded a 40 per cent increase in the number of people receiving multipurpose cash between 2019 and 2020. In the Central African Republic, which recorded a 58 per cent increase in CVA recipients between January and August 2020 as compared to the same period in 2019, adapting to Covid‐19 was a significant driver. In Jordan, approximately 85,000 refugee families received support from the Covid‐19 emergency cash response, with a total of USD 44.3 million being delivered in 2020 (Dürr, 2021).

### Adequacy

CVA expansion has had to operate within the limits of available funding, which has not risen in line with needs (Development Initiatives, 2021). This affects the coverage and sufficiency of the assistance received. By way of illustration, 74 per cent of surveyed UNHCR recipients across 13 countries reported that they were unable to meet more than one‐half of their basic needs (UNHCR, 2020). Challenges to accessing financial support have also been compounded by the impact of Covid‐19 on remittances, with the World Bank (2021) projecting a 14 per cent decline in global remittances by the end of 2021.

### Adaptations and innovations in CVA programming

Cash assistance has worked well partly part due to flexibilities in various dimensions of the delivery chain. The response has necessitated some *changes and innovations in targeting* to expand coverage, including to new target groups or locations (Ground Truth Solutions, 2020b). For example, GiveDirectly was involved in developing or expanding targeting mechanisms for the large‐scale identification of households in need, without sending outsiders into communities for the purpose of enrolment (Blumenstock et al., 2021). These changes included a machine learning‐based targeting model that uses cellular telephone data to estimate poverty levels in Togo (Raftree and Kondakhchyan, 2021b), and list‐based targeting model, leveraging partnerships with community‐based organisations, NGOs, mobile network operators, and government agencies (Interview, Nawar, 2021).

In Colombia, the impact of lockdown on livelihoods compounded already tenuous access to housing among Venezuelan migrants and refugees. To address the resulting homelessness crisis, Mercy Corps and consortium partners targeted households either already evicted or at risk of eviction with a lump sum payment equivalent to two monthly transfers to support housing access, and then assessed them for further support using established criteria. In Ecuador, UNHCR expanded the eligibility criteria for cash assistance given the higher number of people requiring support (Interview, Steinberg, 2021). In northwest Syria, GOAL provided in‐kind assistance during quarantine and a one‐month cash transfer on completion, to help cover lost income, to people referred by health facilities after a positive Covid‐19 test (Interview, al Manla, 2021).

Mounting needs at the household level and lockdown‐related impacts on markets meant *adjustments were required to transfer values and the number of payments provided*. In Gaza, Mercy Corps offered top‐ups for hygiene supplies. In Iraq, border closures affected supply chains, driving up commodity prices, prompting agencies to increase the transfer value. In Syria, GOAL, in partnership with the International Organization for Migration, responded to the rise in the price of soap early in the pandemic with soap in‐kind alongside CVA. Once the price of soap stabilised, it was incorporated into the cash transfer value (Interview, O'Malley, 2021).

It is also important to highlight that some ongoing CVA programmes were negatively affected by Covid‐19. For example, Ground Truth Solutions (2020a) reported that 80 per cent of respondents to a survey in Somalia in September 2020 had seen their transfers either decrease or stop since the onset of the pandemic. This was attributable in part to some conditional cash interventions being suspended, such as cash for work or training activities that entailed people gathering (Ground Truth Solutions, 2020a).

The *imperative to ensure recipient and staff safety*, often through remote operations, was the driving factor in most adaptations. Transmission risks and urgent needs saw *agencies suspend conditionalities for CVA* in some places. In South Sudan, for instance, GOAL's food security and livelihoods programming switched from community asset building to providing basic needs assistance (Interview, Abdissa, 2020). Many agencies adjusted the frequency of payments, collapsing or ‘front‐loading’ multiple transfers into a single lump sum to minimise contact (including trips to automated teller machines (ATMs) or other cash‐out locations) and to support access to basic needs. Organisations also staggered payments to avoid overcrowding at ATMs, increased the number of ATMs or payment points, used mobile ATMs, and employed safety and hygiene protocols at ATMs and other distribution and collection points to reduce transmission risks (Dürr, 2021). There were also cases of Covid‐19 safety messaging being communicated at distribution and collection points (Chadha, Kipkemboi, and Muthiora, 2020).

## Remote programming and the increased digitalisation of humanitarian CVA

A central feature driving the use of CVA has been its *suitability and adaptability for remote delivery*. Much of this has occurred through digital payment solutions that reduce the need for physical contact and as such, have often been perceived as decreasing transmission risks. The pandemic has accelerated the use of digital platforms for CVA delivery by many humanitarian organisations and pushed forward the development of new tools and processes, including new partnerships with the private sector. This section explores the key characteristics, enabling factors, and risks associate with the increased digitalisation of CVA.

### Regulatory shifts to encourage the uptake of digital delivery mechanisms

Governments have played a fundamental role in encouraging the shift to digital payments. For example, many removed barriers to the uptake of mobile money by changing regulations. Major regulatory shifts, including fee waivers and increasing transaction and balance limits, are captured in the Covid‐19 response tracker of the GSMA (Global System for Mobile Communications) (Chadha, Kipkemboi, and Muthiora, 2020). Know Your Customer (KYC) requirements can limit digital payment options if people lack formal identification to register for bank accounts, SIM (subscriber identity module) cards, or mobile money services. Consequently, some governments chose to relax KYC requirements as a temporary measure to respond to the pandemic (Lowe, Yongo, and Corbin, 2021). In Ghana, Kenya, Rwanda, and Zambia, these steps have been made permanent (Chadha, Kipkemboi, and Muthiora, 2020).

The Central Bank of West African States also played a part in promoting digital payments during the pandemic, by pressing service providers to reduce transfer costs (Interview, Amougou, 2021). However, fee reductions may prove challenging to service providers in the long term and finding a balance between accessible financial services and sustainable business models will be essential in the post‐pandemic transition (Theodorou, 2020).

### Going remote: transferring programming processes to virtual and telecommunications platforms

Technological options have also been utilised for communicating with recipients, supporting remote recipient identification, registration, and verification, and data collection and monitoring mechanisms. At the start of the pandemic in early 2020, organisations rapidly shifted processes on to telephone and virtual platforms as far as was feasible, including social media, SMS (short message service), video calls, and WhatsApp. This process has frequently built on or extended existing mechanisms; however, it has also required that organisations simultaneously develop staff capacities to manage both new ways of working and significant increases in demand via telephone and online channels. For instance, in Colombia, Save the Children experienced a 300 per cent rise in calls to the free hotline that it runs on behalf of a cash consortium (Save the Children Colombia, 2021).

Digital tools have increasingly been used to facilitate remote programming, such as to send codes to enable cash redemptions, and to remove the need for paper‐based signatures when confirming the receipt of transfers. Remote verification mechanisms have also been used more and more. In Honduras, Catholic Relief Services (CRS) worked with Telerivet to pilot remote verification via SMS to confirm household identities prior to cash disbursements (Interview, Weatherall, 2021).^3^

Remote data collection, monitoring, and accountability mechanisms have become the default in many organisations during the pandemic. There are pros and cons, but the general sense seems to be that these approaches are ‘good enough’ to enable programming to continue. The enforced switch to remote data collection afforded useful learning. For example, GiveDirectly was surprised to find that the data quality from telephone‐based surveys was often as high as in‐person surveys, and reported that it is likely to adopt a hybrid (remote and face‐to‐face) approach in the future (Interview, Nawar, 2021).

### Working with private sector providers: adaptations and partnerships

Adapting and scaling up digital payments has hinged on strengthening collaboration between humanitarian and private sector actors. For instance, the GSMA has observed better partnerships between humanitarian organisations and mobile money providers, based on open communication and greater alignment of expectations (Interview, Casswell, 2021). In Burkina Faso, the CWG reports that support from financial service providers (FSPs) to adapt platforms in accordance with restrictions has been essential in enabling the scaling up of assistance. In the West Africa region more broadly, the Covid‐19 response has stimulated more dynamic engagement between FSPs and humanitarian actors (Interview, Klein, 2021).

In some cases, the Covid‐19 operating environment meant that different payment mechanisms and FSPs had to be arranged. For example, Save the Children Colombia (2021) was using debit cards and ATMs, but the latter quickly emptied as lockdown commenced. Requiring a reliable alternative, it partnered with Efecty, a national payment service provider with more than 10,000 distribution points that also delivers governmental social assistance.^4^ In Ecuador, strict KYC regulations meant that, pre pandemic, cash assistance for refugees was largely provided through physical distributions. Fortunately, UNHCR's partner was already in discussions with an FSP to find a solution to its cash programme before Covid‐19 hit, which accelerated implementation; some technical issues affecting scale up had to be addressed in real time, but overall, there were no significant gaps or delays in the response (Interview, Steinberg, 2021).

Pre‐existing systems and partnerships have helped adaptation processes. CARE Somalia, with support from the GSMA's Mobile for Humanitarian Innovation programme, was in the process, before the pandemic, of rolling out a biometric verification system using voice identification for mobile money enabled cash assistance, in partnership with Telesom. This system was useful during the crisis because verification, which had previously been conducted in person, could be done entirely remotely using Voice ID (Interview, Mebur, 2021). In South Africa, the Covid‐19 response greatly increased the use of major retailers’ pre‐existing voucher systems for humanitarian purposes. New voucher systems have also been developed there, such as by Joint Aid Management (JAM) International, using SMS (Interview, Otieno, 2021).

### Digital systems and risks

Countries that had digital identity systems before the pandemic, such as India, Peru, and Thailand, were in a better position to deliver targeted large‐scale support (Davidovic, Prady, and Tourpe, 2020). This enabled more than 200 million people in India to receive cash transfers within a week of lockdown, although the lack of a unified social registry was a constraint to the targeting of specific groups, including migrant workers. The state of Bihar set up the Corona Assistance Programme to provide cash transfers to stranded migrant workers, using a ‘digital first’ application, verification, and payments approach. In excess of two million people received assistance. Analysis also reveals, though, that payment mechanisms matter for access, and here, bank accounts may not have been the best option given mobility restrictions, while ‘[d]igital first programs often ignore people at the bottom of the digital pyramid with the least digital capacity’ (Mukherjee, 2020, p. 6).

There is a real risk of exclusion due to the ‘digital divide’ that may not be receiving enough attention (Interview, Taetzsch, 2021). The Collaborative Cash Delivery (CCD) Network is working to address this exclusion challenge by increasing awareness and generating context‐appropriate solutions to improve digital literacy and access (Interview, Taetzsch, 2021). CARE's review of its CVA programming during the pandemic highlights the importance of understanding the context in identifying appropriate digital tools and providing associated support. Doing so requires, inter alia, a solid gender analysis (Dan Aoude and Janoch, 2021), as an estimated 35 per cent of women do not have a bank or mobile money account (Montoya‐Aguirre, Ortiz‐Juarez, and Santiago, 2020). There is heightened demand for toolkits such as GSMA's *Connectivity, Needs and Usage Assessment* ^5^ to help organisations comprehend better mobile telephone access, usage, preferences, and digital skills among populations of concern in a robust and standardised manner (Interview, Casswell, 2021).

The increase in digital cash has also stimulated an increasing focus on data risks and responsibility. This has included questions about the potential challenges posed by digital delivery with respect to humanitarian principles, such as who benefits from ‘going cashless’—the recipients or the service providers?—and the opportunities for surveillance and discrimination (Currion, 2021). These matters are the subject of intense discussion, informed by increasing recognition that humanitarian actors need to understand better and mitigate the risks that digital payments entail, including through improved guidance, tools, and research, such as the CaLP's *Data Responsibility Toolkit and Case Studies* (Raftree and Kondakhchyan, 2021c).

## Expansion through social protection systems and linkages with humanitarian response

‘Many countries [have responded] at a speed and to a level of adequacy and coverage that were unheard of prior to the pandemic—and far superior to anything that a humanitarian system alone could address’ (Interview, Barca, 2021). By the end of December 2020, 215 countries and territories had planned, introduced, or adapted social protection measures in response to Covid‐19 (World Bank, 2020). Cash assistance has accounted for more than one‐half of this programming globally. Estimates suggest that 17 per cent of the world's population received cash through social protection systems in 2020 and at least USD 800 billion was invested in payments and programming (World Bank, 2020). Generally speaking, the key factors limiting flexibility and innovation have been funding, political will, and, critically, the guidance, policy, programming, and operational systems that existed prior to the outbreak of Covid‐19.

Encouragingly, there have been increasing *attempts to respond by linking and coordinating national social protection systems and humanitarian CVA*. Linkages have varied according to the maturity of the national social protection system, the geographical focus of the specific national crisis, the capacity of the state, and the current role of humanitarians. While much of this expansion has been government‐led, or external actors have played only an auxiliary or advocacy part, the role of donors and humanitarian actors has been more significant *in many poor or fragile contexts*, where national systems and leadership are still emerging. Commitments to linking were already in place (Smith, 2021) and the pandemic has provided useful experience, examples of innovations, and deeper insights into challenges.

### Inadequate response

There are clear disparities between countries in terms of coverage, adequacy, duration, and timeliness of response. Looking at the World Bank's real‐time analysis of social protection spending, Gentilini et al. (2021a) state that in ‘low‐income countries, spending per capita amounted to a scant average of $6 per capita, which is 87 times lower than in high‐income countries’. Oxfam found that ‘eight out of 10 countries did not manage to reach even half of their population – women are more likely to be left out of any direct support’ and ‘most of the benefits provided in low‐ and middle‐income countries have been both tiny and temporary’ (Oxfam International, 2020b, p. 3).

### New caseloads

The pandemic shifted patterns of vulnerability and revealed differences within countries. Economic contraction has created new target groups that ‘will not necessarily be the same as either the usual social protection caseload or the target population for “business as usual” humanitarian assistance’ (Wylde, Carraro, and Mclean, 2020, p. 1). The typical focus of social assistance of many nascent or semi‐mature social protection systems has been the extremely poor and vulnerable, or the labour constrained, and those mostly in rural areas. The new caseload of informal, often urban, workers belong to what is described as ‘a “missing middle” that is covered by neither social assistance nor social insurance’ (Franciscon and Arruda, 2020, p. 1). In the DRC, for example, aid has normally centred on the east of the country, but the economic impacts of Covid‐19 were felt strongly in Kinshasa, the capital, and other urban areas.^6^ Other groups have also been disproportionately affected by the crisis or excluded from responses, including women and girls (Cochran et al., 2020). Refugees, displaced persons, and low‐paid migrant workers have also been hit hard, as they were often employed (if at all) in the most informal and insecure parts of labour markets and are mostly ineligible for national assistance programmes (Dürr, 2021; Little, McLean, and Sammon, 2021).

### A nuanced range of responses through social protection and humanitarian systems

The Covid‐19 crisis has seen governmental, developmental, and humanitarian actors innovating across a programming spectrum; ‘one of the significant [resulting] changes to CVA delivery has been the constant reflexions and willingness for linking both types of responses’ (Interview, Gomez, 2021). Elaborating on this theme, some approximate typologies and trade‐offs emerge (Interview, Barca, 2021):

- *Courageous choices—hard to sustain?* Often led and delivered by national governments, countries such as Colombia, Indonesia, Namibia, Pakistan, South Africa, and Togo expanded coverage to groups newly in need of assistance. Critically, existing systems were frequently the basis of the response and innovations were possible precisely because of *prior ‘ecosystem’ investments* (UNECA, 2020; McCord and Rutkowski, 2021). The Government of Pakistan, using its own fiscal resources, launched the Ehsaas Kafaalat programme on 1 April 2020 (Lone, Shakeel, and Bischler, 2021), based on Benazir Income Support Programme mechanisms and complemented by technological innovations for registration and enrolment. Coverage and timeliness were good: five million existing recipients received extra benefits; and 11.9 million temporary new recipients were added—7.3 million were enrolled in the first week. Trade‐offs, inevitable given the speed, model, and resources, were low benefit amounts and a lack of inclusion of women and minorities, owing in part to dependence on mobile telephone technology.
- *Continuing steady expansion over time*. Some governments accelerated ongoing social protection expansions, but de‐prioritised timeliness in favour of increasing coverage gradually. In Nigeria, a new programme for informal workers and vulnerable groups in urban areas was only announced in January 2021 (Lowe, Yongo, and Corbin, 2021). Its Covid‐19 Rapid Response Register cash transfer initiative complements existing platforms under the World Bank‐supported National Social Safety Nets Project, but makes innovative use of data usage for targeting. Similarly, the Government of Uganda led Covid‐19 mitigation efforts but struggled to expand existing schemes, establish new ones, or rapidly negotiate new financing from donors, delaying the response and providing inadequate coverage (Doyle, Hudda, and Marzi, 2021).
- *Adaptation but no expansion*. Other governments made a political choice not to create a new caseload, instead making minor adjustments to existing programmes. Examples include Ethiopia and Ghana. Ethiopia quickly issued guidance to ensure the resilience of its Productive Safety Net Programme in key rural and urban areas, suspending the public works requirements, delivering payments early, and combining several payments (Bischler, Asheber, and Hobson, 2020). However, unwieldy coordination meant that it took months for top‐up payments to be rolled out to 42 and 18 per cent of respective (rural and urban) caseloads. The National Disaster Risk Management Commission, working with humanitarian agencies, reached a further 4.9 million people.
- *Strengthened nascent shock responsive social protection approaches*. In many low‐income, fragile, and conflict‐affected states, government leadership is still emerging. As a result, innovation characterised the roles played by humanitarian and development actors, interfacing and coordinating with governmental cash‐based Covid responses—and with each other—to build on the recent wave of ‘shock responsive’ social protection praxis (Interview, Barca, 2021). In Madagascar, humanitarian and social protection actors built on previous collaboration to coordinate and fund a national cash emergency response to Covid‐19, the *Tosika Fameno* (filling the gap) programme (Interview, Barca, 2021). While government‐led and linked to national systems, the *Tosika Fameno* ensured alignment of partners’ responses (including those of international NGOs, the United Nations Development Programme, and the World Food Programme (WFP)) with that of the government. Insights from this process are now feeding into policy priorities, including joint work on a ‘national recovery strategy’. In Malawi, humanitarian and development partners supported the government in developing a Covid‐19 response master plan, harmonising cash interventions via links to humanitarian and social protection coordination platforms (Smith, 2021; see also Interview, Barca, 2021).

### Role of donors

Donors have made efforts to incentivise closer cooperation between humanitarian cash and social protection systems (Smith, 2021) by bringing together previously separate departments and funding streams. The United Kingdom's Foreign, Commonwealth and Development Office, Germany's Gesellschaft für Internationale Zusammenarbeit (GIZ), and Australia's Department of Foreign Affairs and Trade funded the SPACE (Social Protection Approaches to Covid‐19: Expert advice helpline) team, which has generated a wealth of guidance and reports and provided tailored country advice. Meanwhile, ECHO has ‘been working with colleagues from the EU delegation in various countries through technical assistance, such as expanding shock‐responsive social protection to address new vulnerabilities in urban areas or implementing development funding where our partners can respond more rapidly. Piloting innovations and advocacy towards development actors and national authorities have been central to ensure linkages between cash transfers and social safety nets’ (Interview, Pelly, 2021).

### Flexible funding models

Nearly all agencies have reported rapid repurposing of and innovations in funding to respond to the pandemic (Stade, 2020; Dürr, 2021), including several flexible funding models that accord humanitarian actors better access to development financing or systems. In Ecuador, the WFP channelled funds through the national social protection system; in Vanuatu, a multi‐donor trust fund was set up to disperse humanitarian or social protection assistance. The World Bank's role in providing funding and technical advice and promoting flexibility in the use of large‐scale social assistance schemes globally for the pandemic response has been important. New funding models that reach new combinations of actors with revised incentives permit better response frameworks to address long‐term and recurrent crises. The WFP noted how Covid‐19 had accelerated funding from international financial institutions (IFIs) to unprecedented levels, which could double or triple in coming years. Greater flexibility has also emerged in terms of who pays whom: ‘2020 has been a milestone year for Government‐to‐Person [G2P] payments worldwide, as well as for [the] WFP's role in supporting governments and IFIs in large‐scale service provision and G2P cash delivery’ (Interview, WFP, 2021).

### Trust makes a difference

Joint adaptations have worked best in contexts where collaboration had deeper roots, such as in Madagascar and Malawi. The United Nations Children's Fund (UNICEF) underlined that, ‘prior engagement with the governments on routine social protection is crucial in building trust and developing strong working relations that can be leveraged in emergency response’ (Interview, UNICEF, 2021).

International NGOs have played important roles in this respect, even though much of the social assistance response to Covid‐19 has been channelled through governments or via United Nations (UN) agencies (Interview, Pelham, 2021). UNICEF stated that ‘an additional 1.9 million households were reached using parallel systems in partnerships with local financial service providers and civil society organisations’ (Interview, UNICEF, 2021). Kathryn Taetzsch of World Vision International emphasised that international NGOs continue to play a critical part in ‘bridging the gap’ by sharing their experience in supporting community engagement, bringing about greater accountability and filling gaps—particularly in ‘last mile’, hard‐to‐reach areas—and through their contribution to national system strengthening (Interview, Taetzsch, 2021). Amador Gomez of Action Against Hunger reported that her organisation has made ‘a positive and large effort … in strengthening coordination [with authorities] in countries such as Mali, the Occupied Palestinian Territories, and Ethiopia, or by filling gaps to cover specific affected populations like in [the] Philippines and building capacity on management of payments and community mobilisation in Nigeria’ (Interview, Gomez, 2021). Killen Otieno of JAM pointed out that ‘an unplanned outcome was that we got the government [of South Africa] to play a leading role [in] coordinating both with the national NGOs and with the private sector. That synergy is something that we should capitalise on to build long lasting partnerships’ (Interview, Otieno, 2021). In some contexts, international NGOs assumed critical roles that hint at future engagement based on intellectual capital. For instance, Togo's rapid rollout of a humanitarian cash transfer to those most in need came from a partnership between the government, GiveDirectly, the World Bank, and other actors (Blumenstock, 2021).

### Flows of expertise have gone both ways

In Jordan, the World Bank, UNICEF, and the WFP provided technical support to the National Aid Fund, drawing on expertise honed during the refugee response to adapt rapidly and develop systems to cover newly vulnerable members of the population. In Lebanon, the model of social safety net support being proposed to the government by the World Bank builds on the CVA programme for refugees.

### What next?

Although encouraging, the social protection—and linked humanitarian response— is unlikely to be sufficient or sustained (McCord and Rutkowski, 2021). Valentina Barca of SPACE described much of the social protection response to the pandemic as inadequate to address what is likely to be an L‐shaped recovery, where growth falls and does not return for years (Interview, Barca, 2021). Despite rapid expansion and innovation, many programmes are temporary, with low coverage and transfer values. Pakistan's response via the Economic Coordination Committee, for example, amounted to a one‐off payment equivalent to USD 75, and Namibia's exemplary use of an integrated national registry to create a rapid response (72 hours) self‐registration system entailed a one‐off payment of USD 40.60 (UNECA, 2020). Such small, one‐time payments will be of limited use given the duration and economic impact of the crisis. Most critically, social protection programmes and humanitarian responses are vulnerable to shortfalls in funding from, and the political will of, governments and donors. ‘The crisis is shedding light on longstanding holes in current social protection systems – at the very bottom of the distribution, but also in its “middle”. As such, there are concerns that as the crisis winds down, so will much‐needed social protection programs’ (Gentilini, 2021a).

## Missed opportunity? The role of local organisations in the response

The gap between commitments to localisation on paper and in practice in the humanitarian sector has long been glaring. The signatories to the Grand Bargain (launched during the World Humanitarian Summit in Istanbul, Turkey, in May 2016) committed to channel 25 per cent of all funding to local organisations ‘as directly as possible’ by 2020, but the data show that local actors still struggle to access direct international humanitarian funding (Metcalfe‐Hough et al., 2021). Instead, it is largely distributed via international organisations, which generally retain decision‐making power, leaving local counterparts as implementing partners. Approaches to funding that tend to be short term, ad hoc, and have minimal support costs also do not enable local partners to build the capacity and systems necessary for a quality CVA response (Jodar et al., 2020b).

More than one year on, a key question is whether the Covid‐19 response has created new opportunities for local organisations to access and shape humanitarian aid, or if donors and international agencies have retained their control over funding, strategy, and design? During the early stages of the pandemic, many stakeholders hoped that it might mark a turning point for local organisations. Reflecting this optimism, Gang Karume of Rebuild Hope for Africa said that ‘Covid‐19 presents a unique opportunity to make progress on localisation. Overnight, international actors left. Local actors, who are better integrated in communities, carried on the work. We need to build on this’ (Jodar, 2021).

As the crisis limited movement and travel, international actors often found themselves unable to operate. Larissa Pelham of Oxfam highlighted increased dependence on local organisations, noting that ‘our responses were slower where we did not have those networks in place already’ (Interview, Pelham, 2021). The central role of local organisations in delivering humanitarian assistance during the pandemic is emphasised by those directly involved. Duke Ivn Amin of Jago Nari, a Bangladeshi organisation, observed that local organisations were implementing all of the projects, and that ‘the big difference between local and international organisations is that we will deliver assistance in the field, even where there is a risk to our lives. This is our responsibility and mandate as local organisations’ (Interview, Amin, 2021).

This last point resonates with concerns that predate the pandemic regarding the extent to which localisation ends up down‐streaming risks to local partners (Jodar et al., 2020). This perspective raises the question of whether the Covid‐19 response should be considered as a ‘typical example of localisation’ in that it has underlined disparities in the working conditions and treatment of local partner staff vis‐à‐vis their international counterparts. By way of answer, Sudhanshu S. Singh of Humanitarian Aid International – India remarked that ‘the Covid‐19 response can show the ugly face of localisation. International staff were subject to travel restrictions, and it is understandable that they feared infection, but the same level of respect was not shown for the lives of staff working for local organisations. They were expected to work on the front line, in many cases without social security to fall back on’ (Interview, Singh, 2021).

On a more positive note, there have been constructive elements of the response in terms of working with local organisations. For example, for GOAL, ‘a notable positive effect of Covid‐19 has been the stimulation of collaboration with stakeholders with whom we had not previously engaged’, including with health actors in Syria and Zimbabwe (Interview, O'Malley, 2021). Within Oxfam, the pandemic provided ‘an opportunity to build the capacity and responsibility of local partners to deliver cash [such as in Liberia and the Philippines] and Covid‐19 —positively—has helped cement this already established way of working for us *and has also revealed how far we have to go’* (Interview, Pelham, 2021; see also Oxfam International, 2020a). In Ecuador, UNHCR worked with local organisations in 2020 to address bottlenecks in CVA support linked to protection programming, particularly those with the capacity to reach specific populations, including members of the LGBTIQ (lesbian, gay, bisexual, transgender, intersex, and questioning) community and those vulnerable to gender‐based violence. This included building the capacity of local organisations to deliver cash (Interview, Steinberg, 2021).

It can be argued that the part that local organisations have played during the pandemic demonstrates their capacity to manage resources effectively and demonstrates the importance of their systems and networks at the community level in enabling a rapid and accountable response (Interview, Amin, 2021). One contributor also underscored the significant role of local funding and resourcing in the humanitarian responses to Covid‐19, which is often not duly recognised (Interview, Singh, 2021). However, local actors emphasise that despite stepping up and continuing to deliver, Covid‐19 has not positively affected prevailing power dynamics so far or how these are fundamentally shaped by control of and access to funding. As of May 2020, just 0.1 per cent of total recorded international funding for Covid‐19 responses had been channelled directly to national NGOs (Charter for Change, 2020).

One donor stated that given the need to react quickly early in the pandemic, there was a tendency to stick with existing (mostly international) partners with which relationships were established. However, it was felt that the lessons learned mean that, at a minimum, Covid‐19 will ensure a greater strategic focus on how to support local organisations better in the future (Interview, PRM, 2021).

It is also worth considering the effect that expected reductions in overseas aid and public fundraising, owing to the impact of Covid‐19 on the economies of donor countries, will have on local organisations. With some international NGOs closing certain country offices, this could further cut off critical funding from local partners with no suitable alternative, particularly in countries with more limited local resourcing and fundraising opportunities (Interview, Singh, 2021).

## Will Covid permanently change humanitarian ways of working?

More than a year into the ongoing pandemic, organisations have started to reflect on the key adaptations driven by Covid‐19, and whether these might permanently alter ways of working. Some commentators warn against overstating the extent of the changes to the humanitarian business model due to the pandemic, highlighting that some early predictions of impact have not been borne out, or only to a limited extent (Aly, 2020). For example, Jeremy Wellard of the International Council of Voluntary Agencies noted that responding in the Covid‐19 context has reinforced rather than transformed the role of existing humanitarian coordination structures (Interview, Wellard, 2021). However, he also pointed to the contribution of Covid‐19 in shining a spotlight on existing issues such as racism, colonial structures, and inequalities in the aid system, which reinforce the need for change, particularly concerning localisation (Interview, Wellard, 2021).

Most of the contributors to this paper predicted a mixed picture in terms of longterm changes to CVA. These projections are necessarily closely connected to changes in humanitarian and development/national programming, funding, and systems more broadly and should also be viewed in relation to trends that predate the pandemic.

The expanded use of CVA has increased familiarity and acceptance, which some key informants believe will be a lasting transformation. For instance, it was observed that, ‘despite some early resistance, the Covid crisis has been a game changer in the perception of CVA in West Africa. Previously seen as a complicated, “trendy” topic, it is now widely embraced and acknowledged as an efficient tool to ensure continuity of assistance’ (Interview, Klein, 2021). The broadening of engagement and the development of new partnerships, including with the private sector, is likely to have a lasting effect in some contexts. For example, in South Africa, the development of systems for CVA delivery during the pandemic are predicted to have a long‐term bearing on how aid is distributed, allowing NGOs to leverage well‐established logistics supply chains (Interview, Otieno, 2021).

The widespread loss of income that has been a characteristic of the pandemic, and the corresponding requirement to address a range of basic needs, has contributed to a shift to increased use of multipurpose cash and unconditional transfers. Anissa Toscano of Mercy Corps senses that this is likely to be a long‐term trend, ‘especially as we grapple with the economic hardship worsened by the pandemic in so many communities’ (Interview, Toscano, 2021).

Some key informants identified changes that they anticipate will strengthen programme quality. A PRM official said that the Covid‐19 response has forced programme adaptations that were previously unusual, underlining the importance of having the flexibility to change modalities during a project to address needs better (Interview, PRM, 2021). GOAL stated that it was likely to maintain the more integrated and collaborative cross‐team programming approaches that it has built in several countries of operation, including South Sudan and Zimbabwe (Interview, O'Malley, 2021).

Remote working has been central to pandemic‐related adaptations. In many respects this has hastened an existing trend and underscored the reality that the international humanitarian system was already quite remote by nature, with relatively few actors delivering assistance directly (Interview, Wellard, 2021). One longer‐term outcome could be greater consideration of which activities and interactions do and do not require in‐person contact. For example, CRS remarked that technical support for CVA programming will probably continue remotely in many places, with some tools being adjusted for remote facilitation (Interview, Weatherall, 2021).

Multiple contributors highlighted adaptations and capacities developed for remote programming that are likely to be incorporated into ways of working. Save the Children emphasised the value of institutionalising remote and digital programming options as alternatives in Peru (Save the Children Peru, 2021) and reflected that having developed mechanisms for remote enrolment in Colombia, they may not return to full in‐person processes (Save the Children Colombia, 2021). Within CRS, some continuation of innovative practices is anticipated, such as the use of technology to minimise touch points and increase efficiencies in CVA, although this will not constitute a significant change (Interview, Weatherall, 2021).

Several respondents stressed that despite progress in remote programming, they are keen to return to greater in‐person engagement with communities. ‘While there have been benefits to remote data collection, post Covid, these benefits are unlikely to outweigh the drawbacks in terms of increased potential for data bias, loss of the richness of data and ability to physically verify certain information’ (Interview, O'Malley, 2021).

When it comes to linking social protection to humanitarian response and vice versa, it seems likely that organisations and governments will continue to explore this realm to attempt more systematic action in advance of, and in response to, a crisis (Longhurst et al., 2020). During the pandemic, they expanded existing social protection programmes or developed new ones that built on or used, or ultimately can be linked with, existing systems. Humanitarian actors that have invested in the provision of technical assistance to governments have found this to be a ‘cost‐effective and cost‐efficient way to support crisis‐affected people’ (Interview, UNICEF, 2021). Also encouraging are innovations in data analysis for improved targeting, such as in Namibia or Togo, which are inspiring others elsewhere (Blumenstock, 2021). However, as governments and external actors strengthen interoperability between and within systems, how to manage data responsibly will become increasingly important (Raftree and Kondakhchyan, 2021a).

## Final reflections

It is still too early to draw definitive conclusions on the long‐term impacts of the Covid‐19 pandemic on the implementation and funding of humanitarian CVA, but the modality has so far proven to be an adaptable and scalable means of responding to many needs in a constrained environment. Where the crisis has compelled stakeholders to innovate and shine a light on difficult issues, the opportunities arising should be grasped.

What happens next may be in the hands of donors and IFIs as many countries face unsustainable economic challenges and mounting debt (Oxfam International, 2020b). Unfortunately, it is inevitable that in many countries, many people will not receive adequate support from their government for the foreseeable future. This means that the requirement for humanitarian interventions will persist, although it is probable that the nexus of humanitarian, social protection, disaster risk reduction, and development activities will be increasingly important.

Digitalisation has enabled hundreds of millions of people to receive assistance quickly and safely during the pandemic and the trajectory seems likely to be sustained. Yet, failing to address systematically the digital divide, increase accountability, expand financial inclusion where feasible, and ensure responsible data management may perpetuate the exclusion of those who most need assistance, while generating unacceptable risks.

Predicting what may happen with the localisation of CVA is challenging. However, facilitating greater ownership and action by local actors is crucial to enable more effective and contextually appropriate responses that better represent those affected by crisis. Many of the decisions regarding the future of CVA for the poorest and most vulnerable are influenced by humanitarian and development actors. The pandemic experience to date has shown that adaptation in response is possible. What we need now is a strong commitment to fund and support the positive elements of this reality.

## Appendix

**Table A1:** Key informants

Name	Organisation	Year
Aileen Wynne	GOAL Syria	2021
Alex Nawar	GiveDirectly	2021
Amador Gomez	Action Against Hunger	2021
Anissa Toscano	Mercy Corps	2021
Ciara O'Malley	GOAL	2021
Desire Amougou	Burkina Faso Cash Working Group	2021
Duke Ivn Amin	Jago Nari	2021
Gabriela Prandini	GOAL Zimbabwe	2021
Isabelle Pelly	Directorate‐General for European Civil Protection and Humanitarian Aid Operations	2021
Jenny Casswell	Global System for Mobile Communications	2021
Jennifer Weatherall	Catholic Relief Services	2021
Jeremy Wellard	International Council of Voluntary Agencies (ICVA)	2021
Kathryn Taetzsch	World Vision International/Collaborative Cash Delivery Network	2021
Killen Otieno	Joint Aid Management International	2021
Larissa Pelham	Oxfam	2021
Lucia Steinberg	United Nations Refugee Agency (UNHCR)	2021
Mustafa al Manla	GOAL Syria	2021
Nathalie Klein	Cash Learning Partnership	2021
N/A	Bureau of Population, Refugees, and Migration, US Department of State	2021
N/A	United Nations Children's Fund (UNICEF)	2021
N/A	World Food Programme (WFP)	2021
Semelgen Belay Abdissa	GOAL South Sudan	2021
Sudhanshu S. Singh	Humanitarian Aid International – India	2021
Valentina Barca	Independent/SPACE	2021

**Note:** N/A=not available/applicable. **Source:** authors.

## Data availability statement

The data that support the findings of this study are available on request from the corresponding author. The data are not publicly available due to privacy or ethical restrictions.

## Endnotes

1 As of 15 February 2021, reported funding of the Global Humanitarian Response Plan was USD 3.73 billion of the requested USD 9.5 billion.

2 All of the interviewees quoted throughout this paper gave their consent to be named or to have their organisation named (see Table A1 in the Appendix).

3 For more information, see https://telerivet.com/product/messaging/country/HN (last accessed on 11 November 2021).

4 For more information, see https://www.efecty.com.co/web/pagos (last accessed on 11 November 2021).

5 For more information, see https://www.gsma.com/mobilefordevelopment/resources/the-connectivity-needs-and-usage-assessment-conua-toolkit/ (last accessed on 11 November 2021).

6 Thanks to the anonymous peer reviewer who provided this example.
